# Supplementary material for: The genome sequence of an ichneumonid wasp, Amblyjoppa proteus (Christ, 1791)
Source: Wellcome Open Res. 2025 May 20;10:262. [Version 1] doi: 10.12688/wellcomeopenres.24267.1 (PMC12338164; doi:10.12688/wellcomeopenres.24267.1)
Supplement: Peer review history

# Reviewer report: Reviewer response for version 1

**DOI:** 10.21956/wellcomeopenres.26764.r124770

**Published:** 2025-07-25

**Authors:** Terrence Sylvester

**Affiliations:** 1 University of memphis, memphis, USA

The authors present the genome sequence of the ichneumonid wasp, *Amblyjoppa proteus. *The information provided on the species morphology, behaviour and its distribution is brief but adequate.

 The genome assembly itself appears to be of excellent quality, as supported by standard assembly quality metrics. Authors also report the tissue location they used to generate each sequencing type, which I think is excellent for transparency and reproducibility, particularly for researchers seeking to replicate or adapt similar protocols for related taxa..

 One point that warrants further clarification is the mitochondrial genome assembly. The reported mitogenome size seems larger than expected, considering that insect mitochondrial genomes typically range from approximately 15 to 20 kb. While minor deviations from this range can occur due to duplications or unresolved structural elements, the authors should explicitly comment on this discrepancy. In particular, it would be helpful to clarify whether the assembled mitogenome is complete and circular, and whether it has been fully annotated. Given that the authors used MitoHiFi for this purpose, they should report the annotation results generated by the tool (e.g., number of annotated genes, presence of all 13 protein-coding genes, rRNAs, and tRNAs).

 Additionally, the manuscript would benefit from a summary of annotation quality and completeness metrics for the nuclear genome. While the genome itself is high-quality, the functional annotation is a critical component for downstream analyses. Metrics such as BUSCO scores on predicted gene models would provide a more complete picture of the genome's utility.

 Overall, this is a strong and well-executed genome report. I commend the authors on generating a high-quality resource and recommend publication after addressing the minor issues noted above.

Are sufficient details of methods and materials provided to allow replication by others?

Yes

Is the rationale for creating the dataset(s) clearly described?

Yes

Are the datasets clearly presented in a useable and accessible format?

Yes

Are the protocols appropriate and is the work technically sound?

Yes

Reviewer Expertise:

Genomics

I confirm that I have read this submission and believe that I have an appropriate level of expertise to confirm that it is of an acceptable scientific standard.

# Reviewer report: Reviewer response for version 1

**DOI:** 10.21956/wellcomeopenres.26764.r124207

**Published:** 2025-06-13

**Authors:** Shan Gao

**Affiliations:** 1 Nankai University, Tianjin, China

In the manuscript, the authors have provided the genome sequence of Amblyjoppa proteus with analysis.

 First and foremost, I would like to extend my sincere gratitude to the authors for their valuable contribution to the scientific community.

 However, I feel compelled to offer some constructive feedback regarding certain aspects of the article that could be further refined. I kindly request the authors to consider revising these areas to enhance the overall quality of the manuscript.

 1. The topic need be concise, please avoid to use Latin scientific names. i suggest a new top "The complete genome of an ichneumonid wasp".

 2. In the abstract, authors should clearly report key genomic information. for example, it should be indicated whether this represents the first genome sequenced for this genus or family.  

 If it is not, they should inform readers about the first sequenced genome in that genus or family.

 3. minor suggestions include

 Old: Here we present a chromosomally complete genome sequence for Amblyjoppa proteus, based on a specimen from Wytham Woods,

 New: Here we present a complete genome of Amblyjoppa proteus at the chromosomal level...

 Old: The genome of a specimen of Amblyjoppa proteus (Figure 1) was sequenced using Pacific Biosciences 

 single-molecule HiFi long reads, generating 20.84 Gb from 1.68 million reads, which were used to assemble the genome.

 New: The genome of a specimen of Amblyjoppa proteus (Figure 1) was sequenced using PacBio sequencer,

 generating 20.84 Gb data from 1.68 million reads, which were used to assemble the contigs of the genome.

Are sufficient details of methods and materials provided to allow replication by others?

Yes

Is the rationale for creating the dataset(s) clearly described?

Yes

Are the datasets clearly presented in a useable and accessible format?

Yes

Are the protocols appropriate and is the work technically sound?

Yes

Reviewer Expertise:

genome

I confirm that I have read this submission and believe that I have an appropriate level of expertise to confirm that it is of an acceptable scientific standard.
